# Supplementary material for: Quantum chemistry reveals thermodynamic principles of redox biochemistry
Source: PLoS Comput Biol. 2018 Oct 24;14(10):e1006471. doi: 10.1371/journal.pcbi.1006471 (PMC6218094; doi:10.1371/journal.pcbi.1006471)
Supplement: S1 Table — shows the physiological range of reduction potentials for the major classes of biological electron carriers, as determined by their physicochemical properties and characteristic intracellular concentrations. (DOCX) [file pcbi.1006471.s001.docx]

**Table S1** The range of range of potentials for the most important redox cofactors in biochemistry.

| Electron carrier Type | Minimum E’ | Maximum E’ |
| --- | --- | --- |
| NAD(P)H | -380 mV | -250 mV |
| Glutathione and thioredoxin | -260 mV | -160 mV |
| Quinones and methanophenazines | -400 mV | +400 mV |
| Flavoproteins and free flavins | -500 mV | +100 mV |
| Ferredoxins | -650 mV | +450 mV |
| Cytochromes | -300 mV | +400 mV |

**Table S1** shows the physiological range of reduction potentials for the major classes of biological electron carriers, as determined by their physicochemical properties and characteristic intracellular concentrations. Sources of data for NAD(P)H: [[35,47–51]](https://paperpile.com/c/iMTqC0/rYoIW+q5xNq+tEysD+z0hYV+vtiFi+L4oty); for Glutathione and thioredoxin [[109-112]](https://paperpile.com/c/iMTqC0/TlZ5m+LtebK+48FoP+WfWz2); for Quinones and methanophenazines [[113,114]](https://paperpile.com/c/iMTqC0/OjHEA+4eW9K); for Ferredoxins: [[115-119]](https://paperpile.com/c/iMTqC0/zm8wM+uhJju+6Up75+clJaE+ERhLn); for Flavoproteins and free flavins [[120-123]](https://paperpile.com/c/iMTqC0/9xbHB+ES4TX+q6BBf+0F9ow) and for Cytochromes: [[124–126]](https://paperpile.com/c/iMTqC0/Qks8N+2Ktcx+FPHui)
