# Supplementary material for: Quantum chemistry reveals thermodynamic principles of redox biochemistry
Source: PLoS Comput Biol. 2018 Oct 24;14(10):e1006471. doi: 10.1371/journal.pcbi.1006471 (PMC6218094; doi:10.1371/journal.pcbi.1006471)
Supplement: S2 Table — The model chemistry used consists of density functional theory with the B2PLYP double-hybrid functional, the DefBas-5 Orca basis set (see S3 Table for detailed basis set description), the COSMO implicit solvent, and the D3 dispersion correction. (DOCX) [file pcbi.1006471.s002.docx]

**Table S2:** Linear regression coefficients obtained from calibrating the raw redox potential estimates obtained from the quantum single point energy (SPE) model chemistry consisting of density functional theory with the B2PLYP double-hybrid functional, the DefBas-5 Orca basis set (see Table S3 for detailed basis set description), the COSMO implicit solvent, and the D3 dispersion correction.

| **reaction category** | **slope (**𝛂**)** | **y-intercept (𝛃)** |
| --- | --- | --- |
| G1: Carboxylic Acid to Carbonyl | 0.334 | -382 |
| G2: Carbonyl to Alcohol | 0.408 | -244 |
| G3: Carbonyl to Amine | 0.258 | -232 |
| G4: Alcohol to Hydrocarbon | 0.330 | -57 |
