# Supplementary material for: Quantum chemistry reveals thermodynamic principles of redox biochemistry
Source: PLoS Comput Biol. 2018 Oct 24;14(10):e1006471. doi: 10.1371/journal.pcbi.1006471 (PMC6218094; doi:10.1371/journal.pcbi.1006471)
Supplement: S3 Table — The notation SV(xxx/yyy) refers to the SV basis set with polarization functions xxx and diffuse functions yyy. (DOCX) [file pcbi.1006471.s003.docx]

**Table S3:** A detailed description of the Default Basis (DefBas) sets in Orca version 3.0.3

| **Default-Basis** | **H** | **Main-Group** | **Transition-Metals** |
| --- | --- | --- | --- |
| 1 | SV | SV | TZV |
| 2 | SV | SV(d) | TZV(P) |
| 3 | TZV | TZV(d) | TZV(2pf) |
| 4 | TZV(p) | TZV(2d) | TZV(2pf) |
| 5 | TZV(p/s) | TZV(2d/sp) | TZV(2f/sppd) |
| 6 | TZV(2p/s) | TZV(2df/sp) | TZV(2fg/sppd) |

The notation SV(xxx/yyy) refers to the SV basis set with polarization functions xxx and diffuse functions yyy
