# Supplementary material for: Quantum chemistry reveals thermodynamic principles of redox biochemistry
Source: PLoS Comput Biol. 2018 Oct 24;14(10):e1006471. doi: 10.1371/journal.pcbi.1006471 (PMC6218094; doi:10.1371/journal.pcbi.1006471)
Supplement: S4 Table — The number of available experimental values for each reaction category is indicated in parentheses. MAE = Mean Absolute Error; R2 = coefficient of determination. The quantum model chemistry uses the double hybrid functional B2PLYP with the DefBas-5 default Orca basis set (see S3 Table for detailed basis set description), the COSMO implicit solvent, and the D3 dispersion correction. While the Pearson r can range from -1 to 1, R2 can take on any negative value. A prediction method with the same accuracy as the mean predictor (a constant model that always predicts the mean value of the experimental data) has a value of R2 = 0; negative values of R2 indicate prediction accuracies that are worse than the mean predictor. (DOCX) [file pcbi.1006471.s004.docx]

**Table S4:** Prediction accuracy of the quantum chemistry, molecular fingerprints, and group contribution method modeling approaches. The number of available experimental values for each reaction category is indicated in parentheses. MAE = Mean Absolute Error; R^2^ = coefficient of determination. The quantum model chemistry uses the double hybrid functional B2PLYP with the DefBas-5 default Orca basis set (see Table S3 for detailed basis set description), the COSMO implicit solvent, and the D3 dispersion correction. While the Pearson r can range from -1 to 1, R^2^ can take on any negative value. A prediction method with the same accuracy as the mean predictor (a constant model that always predicts the mean value of the experimental data) has a value of R^2^ = 0; negative values of R^2^ indicate prediction accuracies that are worse than the mean predictor.

|  | **G1 (n=8)**  Carboxylic Acid to Carbonyl | **G2 (n=59)**  Carbonyl to Hydroxycarbon | **G3 (n=23)**  Carbonyl to Amine | **G4 (n=15)**  Hydroxycarbon to Hydrocarbon |
| --- | --- | --- | --- | --- |
| **Quantum Chemistry** | MAE = 45 mV  Pearson r = 0.43  R^2^ = 0.19  No. params. = 2 | MAE = 31 mV  Pearson r = 0.59  R^2^ = 0.35  No. params. = 2 | MAE = 17 mV  Pearson r = 0.70  R^2^ = 0.49  No. params. = 2 | MAE = 34 mV  Pearson r = 0.45  R^2^ = 0.21  No. params. = 2 |
| **Molecular Fingerprints** | N/A | MAE = 28 mV  Pearson r = 0.65  R^2^ = 0.41  No. params. = 25 | MAE = 23 mV  Pearson r = 0.55  R^2^ = 0.21  No. params. = 120 | N\A |
| **group contribution method** | MAE = 52 mV  Pearson r = 0.54  R^2^ = 0.17  No. params. = 6 | MAE = 34 mV  Pearson r = 0.48  R^2^ = 0.21  No. params. = 13 | MAE = 31 mV  Pearson r = 0.22  R^2^ = -0.23  No. params. = 5 | MAE = 66 mV  Pearson r = 0.16  R^2^ = -3.39  No. params. = 6 |
