# Supplementary figures and images for: Quantum chemistry reveals thermodynamic principles of redox biochemistry
Source: PLoS Comput Biol. 2018 Oct 24;14(10):e1006471. doi: 10.1371/journal.pcbi.1006471 (PMC6218094; doi:10.1371/journal.pcbi.1006471)

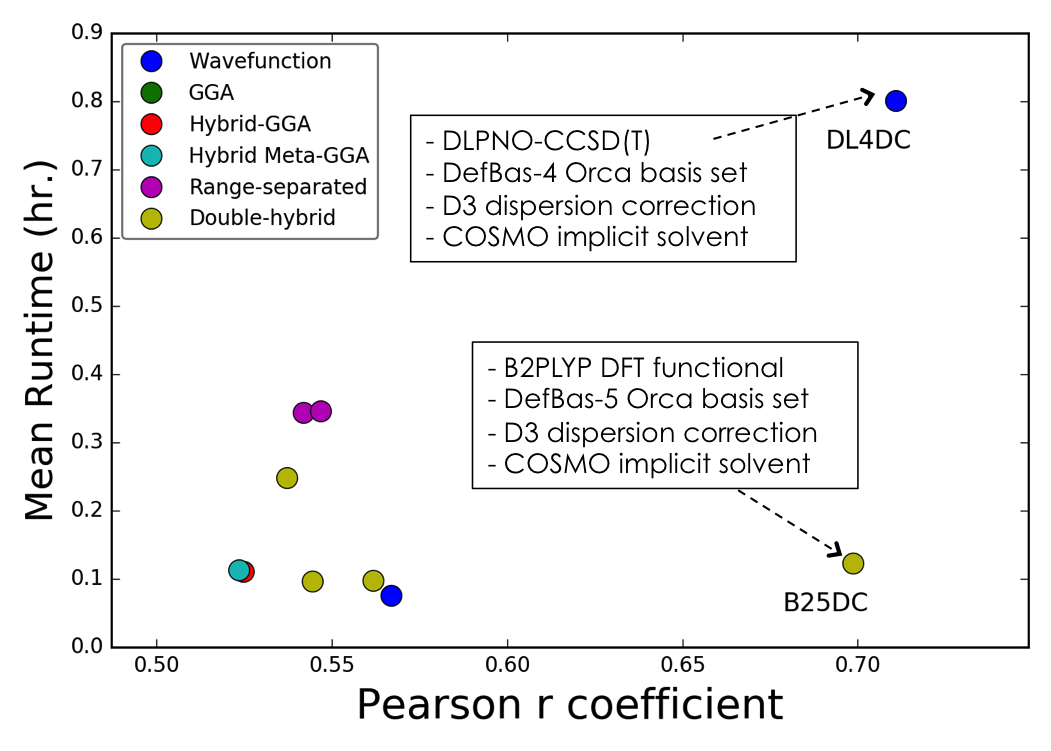

Supplement: S1 Fig — The accuracy measures is obtained from comparing the predicted E′m(pH = 7,I = 0.25) values against available experimental data. Data corresponds to prediction accuracy on the G3 reaction category, which consists of reductions of carbonyls to amines. Mean runtime is calculated over all molecular conformers involved in the simulation of the G3 reaction set with available experimental data. As detailed in section 2.7 “Systematic model chemistry exploration to optimize prediction accuracy”, the SPE model chemistries were obtained from searching over a subspace of possible model chemistries generated from selecting a DFT (or wave function method), a basis set, an implicit solvent model, and a dispersion correction from a total set of: 10 different DFT functionals and 2 wave-function methods, 3 possible basis sets, the option of adding the Conductor-like Screening Model (COSMO) for implicit solvation, as well as the D3 dispersion correction. See Supplementary Dataset 5 for detailed model chemistry descriptions. The option of including or excluding DFT-D3 dispersion correction in the geometry optimization procedure was also considered. (TIF) [file pcbi.1006471.s005.tif]

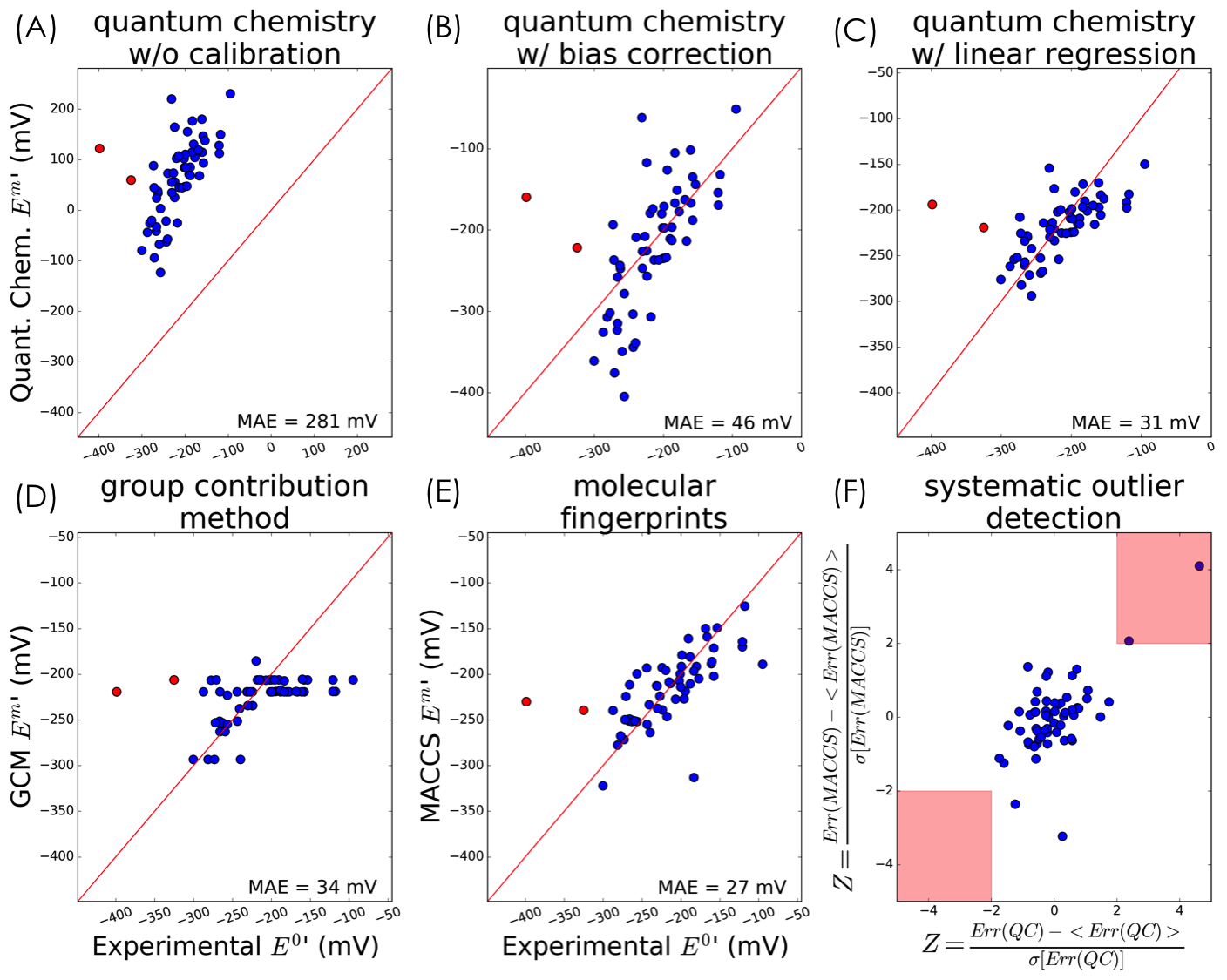

Supplement: S2 Fig — (A-C) Calibrating quantum chemical estimates through linear regression (2-parameters per reaction category) significantly improves prediction accuracy. Quantum chemical predictions were performed using the double-hybrid DFT functional B2PLYP, the DefBas-2 Orca basis set, COSMO implicit solvent, and D3 dispersion correction (S1 Text). Points in red correspond to reactions which consistently appear as outliers across modeling approaches: the indolepyruvate reduction to indolelactate and succinate semialdehyde reduction to 4-hydroxybutanoate (D-E) Prediction accuracy of group contribution method (10 parameters for the G2 category) and molecular fingerprints (166 parameters calibrated with regularized Lasso regression). (F) Scatter plot of normalized prediction errors (z-scores) of G2 reactions for molecular fingerprints and quantum chemistry. The indolelactate dehydrogenase (EC 1.1.1.110) and the succinate semialdehyde reductase (EC 1.1.1.61) reactions have potentially erroneous experimental values. (TIF) [file pcbi.1006471.s006.tif]

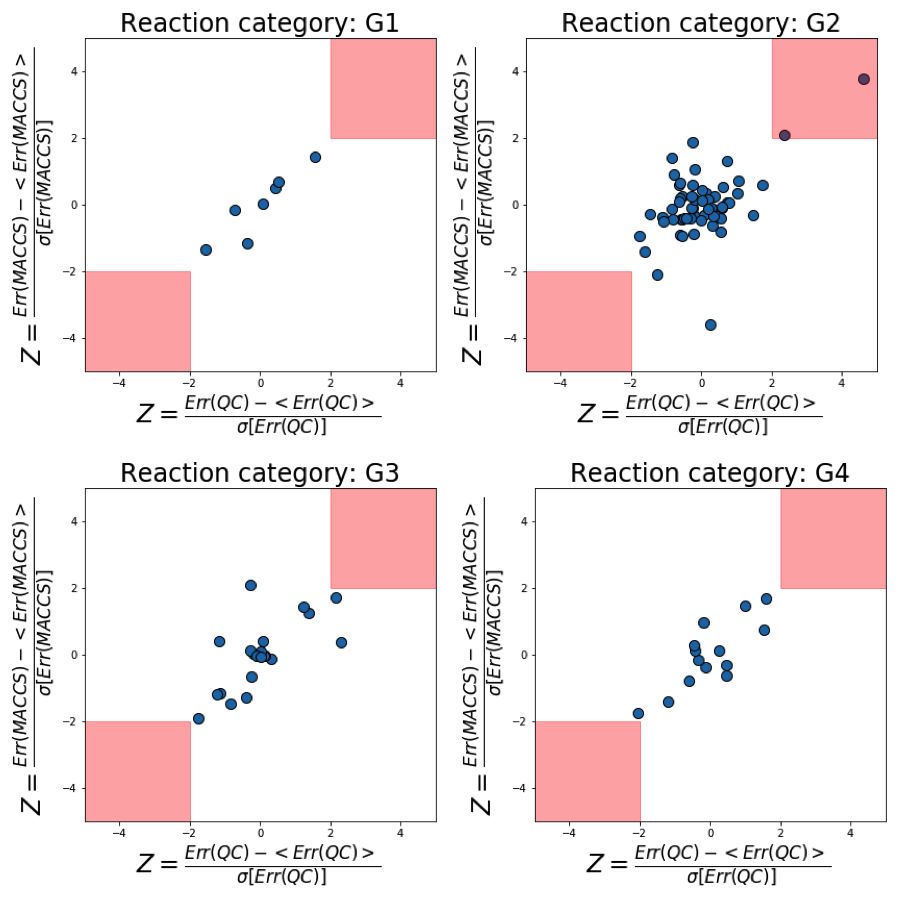

Supplement: S4 Fig — (TIF) [file pcbi.1006471.s008.tif]

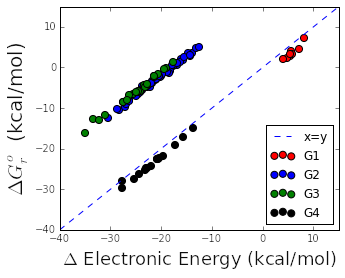

Supplement: S5 Fig — Each redox reaction category is shown in a different color. G1—reduction of carboxyl to aldehyde; G2—reduction of carbonyl (ketone or aldehyde) to hydroxyl; G3—reduction of carbonyl to amine; G4—reduction of hydroxyl to hydrocarbon. ΔEElectronic was obtained from single point energy (SPE) calculations, while ΔGr′o is obtained by additionally including rovibrational contributions to Gibbs formation energy. (TIF) [file pcbi.1006471.s009.tif]

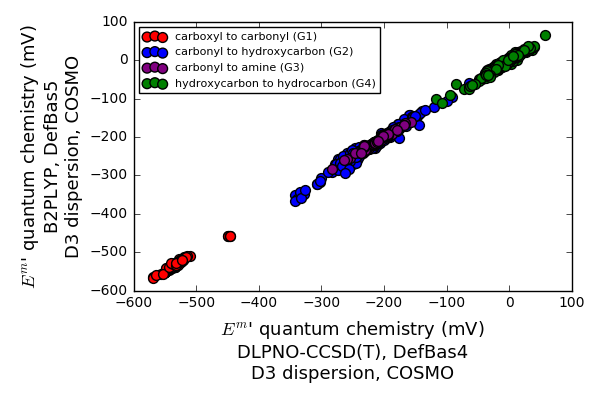

Supplement: S6 Fig — As discussed in the S1 Text, the prediction accuracy of the calibrated model chemistries was evaluated using the experimental data for the G3 reaction category only (to avoid overfitting). The labels refer to the quantum model chemistry used to perform a single point energy (SPE) calculation on geometry-optimized conformers. For both SPE model chemistries, geometry optimizations were performed using B3LYP functional, Orca’s predefined DefBas-2 basis set (S3 Table), COSMO implicit solvent model and DFT-D3 dispersion correction. (TIF) [file pcbi.1006471.s010.tif]

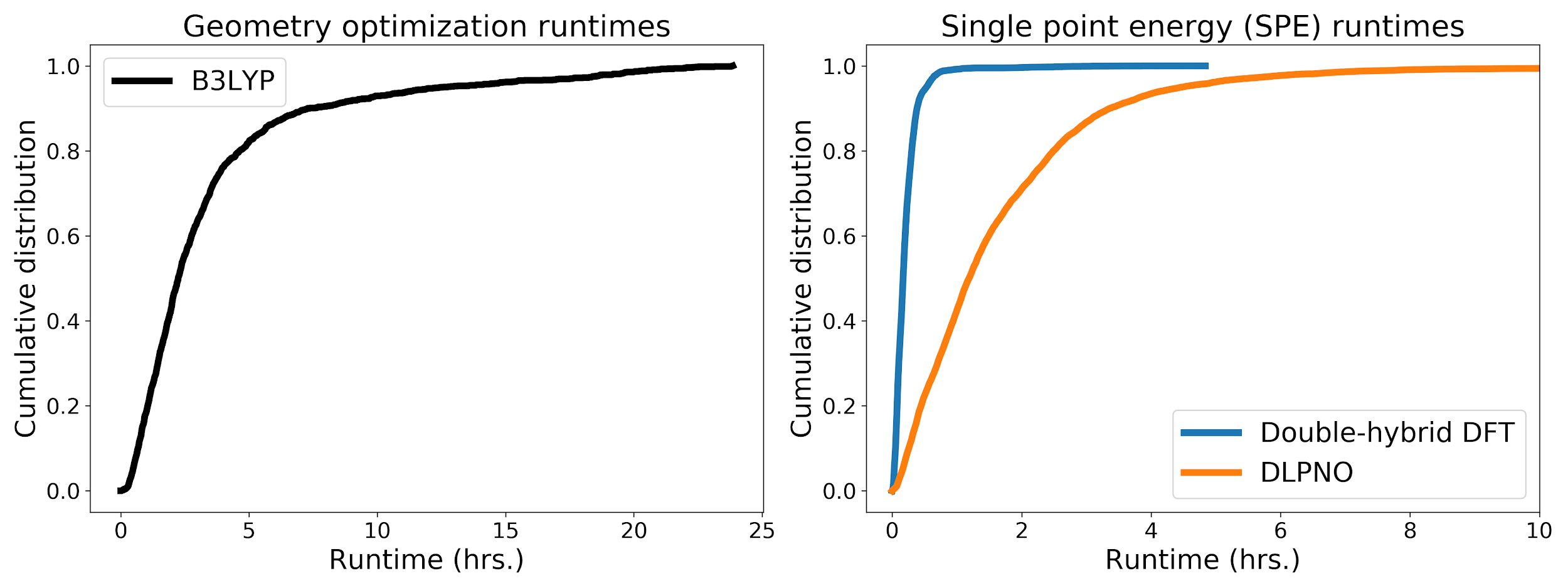

Supplement: S7 Fig — Distributions are over the entire set of molecular conformers used in our study. Geometry optimizations were performed out using DFT, with the B3LYP functional and Orca’s predefined DefBas-2 basis set, as well as the COSMO implicit solvent model (see SI section 2.3). The cumulative distributions of SPE runtimes are shown for the two best-performing model chemistries: the linear-scaling coupled cluster method DLPNO-CCSD(T), with the DefBas-4 Orca basis set (S3 Table), COSMO, implicit solvent, and D3 dispersion correction; and the double-hybrid functional B2PLYP, the DefBas-5 Orca basis set (S3 Table for detailed description), COSMO implicit solvent, and D3 dispersion correction (see SI section 2.7 for further details). (TIF) [file pcbi.1006471.s011.tif]
