# Supplementary material for: Quantum chemistry reveals thermodynamic principles of redox biochemistry
Source: PLoS Comput Biol. 2018 Oct 24;14(10):e1006471. doi: 10.1371/journal.pcbi.1006471 (PMC6218094; doi:10.1371/journal.pcbi.1006471)
Supplement: S3 Fig — Quantum chemical predictions were performed using the double-hybrid DFT functional B2PLYP, the DefBas-2 Orca default basis set, the COSMO implicit solvent, and D3 dispersion correction (S1 Text). Data corresponds to experimental values and predictions at the pH = 7 and I = 0.25 biochemical state. G1: reduction of an unmodified carboxylic acid (-COO) to a carbonyl (-C = O); G2: reduction of a carbonyl to a hydroxycarbon (-COH, i.e., alcohol); G3: reduction of a carbonyl to an amine (-CNH3); and G4: reduction of a hydroxycarbon to a hydrocarbon (-C-C-). (DOCX) [file pcbi.1006471.s007.docx]

**Supplementary Material for “Quantum chemistry reveals thermodynamic principles of redox biochemistry”**

**Table of contents:**

1. Introduction
2. A calibrated quantum chemical approach to estimating biochemical standard transformed redox potentials
   1. Selecting compounds’ major species at pH = 0 for quantum chemical calculations
   2. Obtaining initial geometric conformations
   3. Quantum chemical geometry optimizations
   4. Quantum chemical single point electronic energies to estimate standard chemical redox potentials
   5. Transforming $E^{o}(MS at pH=0)$ to ${E'}^{m}(p=7, I=0.25)$using pKa estimates, the extended Debye-Huckel equation and the Alberty-Legendre transform
      1. Using pKas to get relative energies of different protonation states (species)
      2. Accounting for ionic strength with the extended Debye-Huckel equation
      3. Transforming chemical standard redox potentials to transformed standard redox potentials at pH=7 with the Alberty-Legendre Transform and isomer group equations
   6. Calibration via linear regression against experimental data
   7. Systematic model chemistry exploration to optimize prediction accuracy
   8. Correlation between the difference in quantum chemical electronic energies and quantum chemical standard Gibbs reaction energies.
   9. Computational cost of quantum chemical method
3. Construction of dataset of experimental standard redox potentials
4. Molecular fingerprint and group contribution method estimates of redox potentials
   1. Group contribution method
   2. Molecular fingerprints
5. Redox potentials of redox cofactors in biochemistry
6. Generation of comprehensive database of natural and non-natural redox reactions

**1. Introduction**

Our aim is to predict transformed standard redox potentials at any pH and ionic strength (I) of interest. Biochemical thermodynamics differs from chemical thermodynamics in that - in addition to temperature and pressure - pH and ionic strength are natural variables that need to be specified [[1]](https://paperpile.com/c/iMTqC0/k9fI). At the biochemical standard state, every metabolite exists as an equilibrium ensemble of different species (or protonation states), each with a different charge and number of hydrogen atoms. Experimental data for biochemical thermodynamics consists of apparent equilibrium constants K´ measured at a specified value of pH and I. The apparent equilibrium constants K´ are equilibrium ratios of sums of species. In contrast, chemical equilibrium constants are equilibrium ratios of individual species (protonation state) concentrations. Using a set of pKa values, and the Legendre transform developed by Robert Alberty [[1]](https://paperpile.com/c/iMTqC0/k9fI), standard thermodynamic parameters (K, ΔG^o^, E^o^) can be converted to standard *transformed* thermodynamic parameters (K’, ΔG’^o^, E’^o^) of interest.

Because of challenges associated with accurately simulating organic anions in solution with quantum chemistry [[2,3]](https://paperpile.com/c/iMTqC0/mzspx+kXdZ9), we developed the following pipeline: for every metabolite involved in a redox reaction, we run quantum chemical simulations (geometry optimization and single point energies) to obtain the electronic energies of the major (most abundant) species at pH=0. We use the difference in electronic energies of the major species of products and substrates at pH=0 as an estimate of the corresponding standard redox potential, $E^{o}(MS at pH=0)$. Next, we use empirical pKa estimates, the extended Debye-Huckel equation, and the Alberty-Legendre transform to convert the standard redox potential $E^{o}(MS at pH=0)$ to the standard (standardized to 1 mM) *transformed* redox potential ${E'}^{m}( pH=7, I=0.25)$. To correct for systematic errors in both the quantum chemical predictions and the pKa estimates, we calibrate the resulting ${E'}^{m}( pH=7, I=0.25)$ values against experimental data using linear regression. We perform a separate calibration for each of the four different redox reaction categories.

Below we describe in detail each of these steps. We also describe how we generate group contribution method and molecular fingerprint estimates of redox potentials. Finally, in the last section we describe in detail how we generated a database of ~650 natural and non-natural redox reactions using the KEGG database of metabolic compounds.

**2. A calibrated quantum chemical approach to estimating biochemical standard transformed redox potentials**

**2.1 Selecting compounds’ major species at pH = 0 for quantum chemical calculations**

For each metabolite involved in a redox reaction of interest, we obtain the protonation state corresponding to the major (most abundant) species at pH=0 using the ChemAxon calculator plugin (Marvin 17.7.0, 2017, ChemAxon). Specifically, we use the command “cxcalc majorms -H 0 -f smiles” to obtain the smiles string of the major species at pH=0. Dataset S3 contains the full set of compound names, KEGG compound identifiers, smiles strings (for the major species at pH=0), and charge (for the major species at pH=0) used in this work. Although we chose to use ChemAxon as our cheminformatics software tool based on its wide use in the biochemical thermodynamics community, we performed sporadic testing with two alternative open source cheminformatics tools (OpenBabel and RDKit) and note that these could in principle be used instead.

**2.2 Obtaining initial geometric conformations**

For each metabolite, we generate ten initial geometric conformations. These initial conformers serve as inputs to the quantum geometry optimizations. Conformers were generated using ChemAxon cxcalc calculator plugin, (Marvin 17.7.0, 2017, ChemAxon). Specifically, we use the command “cxcalc conformers -m 10”. Performing geometry optimization over this set of initial conformers allows us to explore several local minima in each molecular potential energy surface. For small metabolites where the cxcalc conformer command generates less than 10 geometric conformations, we limit the quantum chemical calculations to the maximum number of conformers generated.

**2.3 Quantum chemical geometry optimizations**

All quantum chemistry calculations were performed using the Orca software package (version 3.0.3) [[4]](https://paperpile.com/c/iMTqC0/g7A6N). Geometry optimizations were carried out using DFT, with the B3LYP [[5]](https://paperpile.com/c/iMTqC0/toS0w) functional and Orca’s predefined DefBas-2 basis set (Table S3). The COSMO implicit solvent model [[6]](https://paperpile.com/c/iMTqC0/zIIPz) was used, with the default parameter values of epsilon = 80.4 and refrac = 1.33. We also tested the effect of including or excluding DFT-D3 dispersion correction [[7]](https://paperpile.com/c/iMTqC0/dembK) using Becke-Johnson damping [[8]](https://paperpile.com/c/iMTqC0/r6Clg) in the geometry optimization procedure, using the “D3” flag in the Orca input file (Fig S1). For all reaction categories, molecular hydrogen (KEGG compound ID C00282) was used in the substrate side of the reactions (in the direction of reduction). For categories G1 (reduction of carboxylic acid to aldehyde) and G4 (reduction of alcohol to hydrocarbon), the reaction was balanced with an additional water molecule in the product side of the reaction (in the direction of reduction). For category G3 (reduction of carbonyl to amine), an additional ammonia molecule was added to the substrate side of the reaction (in the direction of reduction).

All calculations were run in Harvard’s Odyssey cluster using single cores. Initial orbital guess was set to PModel, and the default TightSCF convergence criteria was set for the self-consistent field iterations. The integral handling flag was set to SCFMode Direct. A example Orca input file for the geometry optimizations is shown below:

! RHF B3LYP D3 DefBas-2 AUTOSTART TIGHTSCF PMODEL OPT

%cosmo epsilon 80.4

refrac 1.33

end

%scf

SCFMODE DIRECT

end

%output

XYZFile true

end

%geom

MaxIter 1000000

end

%pal nprocs 1

end

* xyzfile 0 1 mol_geometry.xyz

**2.4 Quantum chemical single point electronic energies to estimate standard chemical redox potentials**

We used the optimized geometries obtained using DFT as described above as inputs for single point electronic energy (SPE) calculations with different model chemistries (see below). By taking the difference of products’ and substrates’ $E_{Electronic}$values, we obtain $\Delta E_{Electronic}$, which we treat as directly proportional the standard reduction potential:

$E^{o}(MS at pH=0)=-\Delta{G_{r}}^{o}(MS at pH=0)/nF \sim-\Delta E_{Electronic}(MS at pH=0)/nF$,

where n is the number of electrons and F is Faraday’s constant. This is motivated by the empirical observation that there is a strong linear correlation between ΔE_Electronic_ and ΔG_r_^o^ for these systems (section 2.8, Fig S5). Substrate and product conformers were randomly sampled over 30 iterations using a Boltzmann distribution [[2]](https://paperpile.com/c/iMTqC0/mzspx). The average ΔE_Electronic_ across all samples was used as the estimate for the redox potential. All calculations were run in Harvard’s Odyssey cluster using a single core. Initial orbital guess was set to PModel, and the default TightSCF convergence criteria was set for the self-consistent field iterations. An example Orca input file for the single point energy calculations is shown below.

! B2PLYP-D3 DefBas-5 Grid5 RIJCOSX FINALGrid6 TIGHTSCF PMODEL

%cosmo epsilon 80.4

refrac 1.33

end

%pal nprocs 1

end

%MaxCore 2000

* xyzfile 0 1 B3LYP_optimized_geometry.xyz

**2.5 Transforming** $\boldsymbol{E}^{\boldsymbol{o}}\boldsymbol{(MS at pH=0)}$**to** $\boldsymbol{E'}^{\boldsymbol{m}}\boldsymbol{(pH=7, I=0.25)}$ **using pKa estimates, the extended Debye-Huckel equation and the Alberty-Legendre transform.**

Next, we break down into three steps the conversion of standard redox potentials for the major species at pH=0 to *transformed* potentials at pH=7 and ionic strength I=0.25. Following Alberty^1^, we use the notation where the main transformations are applied to Gibbs formation energies $\Delta{G_{f}}^{o}$. However, since (as noted above and in section 2.8) we do not consider translational, rotational and vibrational enthalpies and entropies in our calculations, in practice in our pipeline we apply all the transformations described below to electronic energies of compounds, $E_{Electronic}$.

**2.5.1 Using pKa’s to get relative energies of different protonation states (species)**

Using a set of pKa values from ChemAxon’s cxcalc calculator plugin (Marvin 17.7.0, 2017, ChemAxon), the estimate for the chemical redox potential of the major species at pH=0 obtained from quantum chemistry - $E^{o}(MS at pH=0)$ - can be converted to the chemical potential of any other reference species (protonation state) of interest, e.g. $E^{o}(MS at pH=7)$. The set of relevant protonation states is obtained by computing the acid dissociation constants within the range MIN_PH = 0.0 and MAX_PH = 14.0 with the cxcalc command GetDissociationConstants. The formation energy of the n-th species (protonation state) relative to energy of the major species at pH=0 can be obtained using the pKa’s via the relation

$$\Delta{G_{f}}^{o}(n) -\Delta{G_{f}}^{o}(0) = -RTln(10)\sum_{i=1}^{n} pKa_{i}$$

where pKa_i_ is the pKa of the i-th species, with the zero-th species defined as the major species at pH=0.

**2.5.2 Accounting for ionic strength with the extended Debye-Huckel equation:**

Our aim is to predict redox potentials in the biochemical state at pH=7 and ionic strength I=0.25. Following the treatment by Alberty [[1]](https://paperpile.com/c/iMTqC0/k9fI), we account for the effect of ionic strength on the activity coefficients of ionic species of metabolites by means of the extended Debye-Huckel equation [[9]](https://paperpile.com/c/iMTqC0/pKAZR):

$$ln(\gamma_{i}) = -A{z_{i}}^{2}I^{1/2}/(1+BI^{1/2})$$

where A is 2.91482 L^-1/2^ mol^1/2^ at T = 298.15 K and B is 1.6 L^1/2^ mol^-½^ [[9]](https://paperpile.com/c/iMTqC0/pKAZR). The extended Debye-Huckel equation leads to the following expression for formation energy a species as a function of ionic strength [[1]](https://paperpile.com/c/iMTqC0/k9fI):

$$\Delta{G_{f}}^{o}(I) =\Delta{G_{f}}^{o}(I=0) -A{z_{i}}^{2}I^{1/2}/(1+BI^{1/2})$$

**2.5.3 Transforming chemical standard redox potentials to transformed standard redox potentials at pH=7 with the Alberty-Legendre Transform and isomer group equations:**

Under the Alberty-Legendre transform framework, a new thermodynamic potential - the transformed potential, indicated by the prime notation (´) - is obtained. This transformed potential properly accounts for pH as a natural variable. The process involves two main steps. In the first step, the formation energy of each *individual species* (protonation state, indicated by the index j) is converted into the standard transformed energy of formation at the pH of interest using the following Legendre transform:

$$\Delta{G_{f}}^{o'}[j](pH) =\Delta{G_{f}}^{o}[j] - N_{H}[j]\{\Delta{G_{f}}^{o}[H^{+}]-RTln(10)pH\}$$

Where N_H_ is the total number of hydrogen atoms in the j-th species. Combining this with the expression for the standard formation energy as a function of ionic strength, yields:

$$\Delta{G_{f}}^{o'}[j](pH, I) =\Delta{G_{f}}^{o}[j](I=0)+N_{H}(RTln(10)pH -\Delta{G_{f}}^{o}[H^{+}])-A{{(z}_{i}}^{2}-N_{H})I^{1/2}/(1+BI^{1/2})$$

In the second step, the standard transformed energy of formation of all N species (protonation states) that exist in equilibrium at pH = 7 are combined using what Alberty calls “isomer group equations” into a single *transformed* (or “apparent) formation energy [[1]](https://paperpile.com/c/iMTqC0/k9fI):

$$\Delta{G_{f}}^{o'}(pH, I) = -RTln\{\sum_{j=1}^{N} exp(-\Delta{G_{f}}^{o'}[j](pH, I)/RT)\}$$

These transformed compound energies are then stoichiometrically combined to obtain the transformed reaction energies (and in our case, the estimates for the transformed redox potentials).

Finally, we note that we convert all *transformed* potentials to the millimolar standard state (i.e. assume a concentration of 1 mM for substrates and products). In practice this conversion only affects the energetics of reactions in the G3 category (reduction of carbonyls to amines), because - as noted above - these are balanced with an ammonia molecule as a substrates. This therefore introduces a factor of $RTln(10^{-3})$when converting to the mM standard state.

**2.6 Calibration via linear regression against experimental data:**

As described in the main text Methods section, having transformed the redox potentials to the transformed standard (1mM standard state) state pH = 7 and I = 0.25, ${E'}^{m}( pH=0, I=0.25)$ we performed linear regressions between the quantum chemical estimates and the available experimental redox potentials. We performed a separate regression for each of the four different redox reaction categories considered. The calibration through linear regression aims to correct for systematic errors in both the quantum chemical predictions and the pKa estimates used in the Legendre transform, as well as any other systematic errors introduced by assumptions in our quantum chemical modeling framework. The calibration using linear regression was implemented using the SciKit learn Python machine learning library [[10]](https://paperpile.com/c/iMTqC0/LNfJe). To measure the resulting prediction accuracy, we partition reactions into training and validation sets using Leave-One-Out Cross Validation (LOOCV). Fig S2 (A-C) demonstrates - for reactions in the G2 category (reductions of carbonyls to hydroxycarbons) - the effect on accuracy of calibrating the quantum chemistry predictions of ${E'}^{m}( pH=0, I=0.25)$with one and two parameters, in comparison to the many-parameter predictions of group contribution method and molecular fingerprints (Fig S2, D-E).

**2.7 Systematic model chemistry exploration to optimize prediction accuracy**

In order to optimize prediction accuracy and choose a single point energy (SPE) model chemistry that minimizes prediction error, we performed a systematic combinatorial search over a subspace of different model chemistries. We define an SPE model chemistry as combination of one DFT functional or wave-function electronic structure method, a basis sets, a choice of implicit solvent, and a choice of dispersion correction. We included the following DFT functionals in our search space: PBE[[11]](https://paperpile.com/c/iMTqC0/MrOtX), PBE0[[12]](https://paperpile.com/c/iMTqC0/7SJV3), B3LYP,[[5]](https://paperpile.com/c/iMTqC0/toS0w) M06[[13]](https://paperpile.com/c/iMTqC0/RuhvD), M06-2X[[13]](https://paperpile.com/c/iMTqC0/RuhvD), TPSS0[[14]](https://paperpile.com/c/iMTqC0/xtvv6), wB-97x[[15]](https://paperpile.com/c/iMTqC0/weYKF), CAM-B3LYP[[16]](https://paperpile.com/c/iMTqC0/EhOl0), B2PLYP[[17]](https://paperpile.com/c/iMTqC0/AeYza), DSD-PBEP86[[18]](https://paperpile.com/c/iMTqC0/uSEsx). We also include two wave function electronic structure methods: the domain-based local pair natural orbital (DLPNO) Coupled Cluster method [[19]](https://paperpile.com/c/iMTqC0/PoSUY) and the spin-component-scaled Moller-Plesset perturbation theory (SCS-MP2) [[20]](https://paperpile.com/c/iMTqC0/jku9c). We include three possible basis sets, the “default basis sets”: DefBas-4, DefBas-5, DefBas-6. These “default-basis sets” in Orca version 3.0.3 are based on Ahlrichs basis sets, are of increasing quality, and differ in the polarization and diffuse functions used for hydrogen atom, main-group elements, and transition metals (see Table S3 for detailed description of these Orca default basis sets). We also include the option of adding the Conductor-like Screening Model (COSMO) [[6]](https://paperpile.com/c/iMTqC0/zIIPz) for implicit solvation, as well as the D3 dispersion correction [[7]](https://paperpile.com/c/iMTqC0/dembK).

We performed combinatorial optimization over this subspace of SPE model chemistries using a 1-dimensional greedy search algorithm. At each cycle of the algorithm, all parameter values are fixed except for one, the active parameter, which is allowed to vary across each iteration. For example, in the first cycle, the basis-set, implicit solvent, and dispersion correction are fixed at specific values (e.g. DefBas-4, COSMO, D3). At each iteration within the cycle, a different DFT functional or wave function method is selected. SPE calculations are run with the 4 selected parameters on the full set of reactions with experimentally measured redox potentials. After each SPE calculation with a specific parameter combination, we transform the estimated standard redox potentials to the standard *transformed* redox potentials E^m^’(pH=7, I = 0.25) using pKa estimates, the extended Debye-Huckel equation, and the Alberty-Legendre transform (see sections 2.5.1 - 2.5.3) and calibrate against the set of experimental redox potentials using the linear regression approach described above (section 2.6). At the end of each cycle, the value of the active parameter resulting in highest Pearson correlation coefficient (r) is selected and fixed for the next cycle. Therefore, the objective function of the search is the Pearson correlation coefficient of the calibrated model chemistry with experiment.

To avoid overfitting, we trained the the model chemistry optimization procedure on the experimental data for the G3 reaction category (carbonyl to amine reduction), and validated its accuracy on the rest of the oxidoreductase reaction categories (Table 1 and Table S4). We also tested the effect of a few geometry optimization (GO) model chemistries. Importantly, we found that performing the optimization using a model chemistry without dispersion correction (i.e. B3LYP functional, Orca’s predefined DefBas-2 basis set (Table S3), COSMO implicit solvent model but without DFT-D3 dispersion correction) decreased the prediction accuracy as captured by the Pearson r. Fig S1 shows the average accuracies and runtimes obtained for a set of model chemistries explored (see Supplementary Dataset 5 for model chemistry details). In the end, given its high accuracy and relatively low computational cost, we selected the geometry optimization model chemistry that includes D3 dispersion correction, and an SPE model chemistry that uses the double-hybrid functional B2PLYP [[17,21]](https://paperpile.com/c/iMTqC0/Hj2RS+AeYza), the DefBas-5 Orca [[4]](https://paperpile.com/c/iMTqC0/g7A6N) basis set (Table S3 for detailed description), COSMO [[6]](https://paperpile.com/c/iMTqC0/zIIPz) implicit solvent, and D3 [[7]](https://paperpile.com/c/iMTqC0/dembK) dispersion correction. Table S2 shows, separately for each reaction category, the linear regression coefficients obtained using this model chemistry.

We note that other model chemistries gave similarly high prediction accuracies. In particular, the SPE model chemistry consisting of the linear-scaling coupled cluster method DLPNO-CCSD(T)[[19]](https://paperpile.com/c/iMTqC0/PoSUY), with DefBas-4 Orca [[4]](https://paperpile.com/c/iMTqC0/g7A6N) basis set (Table S3), COSMO [[6]](https://paperpile.com/c/iMTqC0/zIIPz) implicit solvent, and D3 [[7]](https://paperpile.com/c/iMTqC0/dembK) dispersion correction (and DFT geometry optimization that includes D3 dispersion correction), had a similarly high accuracy, but at a significantly higher computational cost. Importantly, the predicted ${E'}^{m}( pH=7, I=0.25)$ values obtained with both model chemistries are highly correlated, and thus our main results (i.e. Figs 2, 3, and 4) are consistent across both of these calibrated model chemistries (Fig S6).

**2.8 Correlation between quantum chemical difference in electronic energies and quantum chemical Gibbs reaction energies.**

As noted above and in Methods, the use of quantum chemical ΔE_Electronic_ as opposed to the Gibbs reaction energy ΔG_r_^o^ to approximate the chemical redox potential is motivated by empirical observation that there is a strong correlation between ΔE_Electronic_ and ΔG_r_^o^ for these systems. Fig S5 shows the linear correlation between ΔE_Electronic_ and ΔG_r_^o^ for all reaction categories. The systematic biases in the ΔE_Electronic_ values for the reduction of carbonyls to alcohols or amines (reaction categories G2 and G3) may be largely explained by the difference in vibrational enthalpies of the substrates’ C=O double bonds and products’ C-O (C-N) single bonds. The systematic error introduced by this approximation is corrected for by the calibration of our quantum chemical simulations against experimental values using linear regression.

In summary, we decided to use ΔE_Electronic_ obtained from quantum chemistry instead of ΔG_r_^o^ as an estimate of the chemical redox potential based on the following three observations: (1) there this strong linear correlation between quantum chemical ΔE_Electronic_ and ΔG_r_^o^, (2) our pipeline already involves calibration against experiment using linear regression (see above), (3) there is significantly lower computational cost in estimating only ΔE_Electronic_ instead of ΔG_r_^o^ with quantum chemistry.

**2.9 Computational cost of quantum chemical methodology.**

Running quantum chemical simulations of metabolites is significantly more expensive than running the group contribution method. While the latter can be run in a personal computer for large sets (hundreds) of metabolites in a matter of minutes, doing quantum chemistry on hundreds of compounds using double-hybrid DFT or DLPNO coupled-cluster requires access to a high performance computing cluster. Fig S7 shows the cumulative distribution of runtimes for the two portions of our quantum chemistry calculations, geometry optimizations and single point electronic energy estimates (SPE). The geometry optimization portion of the pipeline is significantly more expensive, and we parallelized these calculations over 16 cores.

We note that, since we calibrate the raw quantum chemistry predictions against experiment using linear regression, more expensive methods do not necessarily result in higher prediction accuracies. As shown in Fig S1 and Fig S6, while our DLPNO single point energy calculations are on average about 8 times as expensive as our double-hybrid DFT simulations (using the B2PLYP functional), both model chemistries yield similar favorable accuracies. This is why we choose to use the double-hybrid DFT data for most of our analyses.

**2.10 Sensitivity of quantum chemistry approach to modeling choices and parameters.**

In this section, we discuss the sensitivity of the prediction accuracy of our calibrated quantum chemistry methodology to the different modeling choices and parameter values.

As discussed above, we set the ChemAxon major microspecies pH parameter to pH = 0. The important aspect of this choice is that the method needs to obtain the most (fully) protonated microspecies as a “reference microspecies” when performing the quantum chemical calculations. Given typical pKa values of metabolic compounds, the pH = 0 parameter in the major microspecies method ensures that fully protonated microspecies are obtained. In addition, since the large majority of metabolic compounds in our analysis have pKa values higher than pKa~2, the method works robustly when this parameter set to pH=1 or pH=2 as well. However, in previous work [2], we showed that the main source of errors in quantum chemistry estimates of standard Gibbs energies of diverse metabolic reactions comes from running electronic structure calculations on charged, ionic species. That is, the method is very sensitive to inclusion of negatively charged microspecies when running the quantum chemistry. Therefore, running the method with a value of the pH parameter that starts resulting in numerous ionic major microspecies (i.e. pH ≥ 3) will lead to severe downstream prediction errors.

As mentioned, we set ChemAxon’s cxcalc to generate 10 conformers for each metabolic compound involved in a redox reaction, and run quantum chemistry simulations on all such initial geometric conformers. The value of 10 reflects a tradeoff between sampling as large a number of conformers as possible to exhaustively explore the molecular potential energy landscape of each compound, yet satisfying the constraints imposed by available computational resources. For a smaller set of reactions, we tested the accuracy of predictions with fewer conformers. Although in general predictions were robust to running simulations on fewer conformers (down to 5), the predicted values for some reactions were unstable. In addition, although computationally more expensive, we tested a subset of reactions with more than 10 conformers, yet did not observe a significant improvement in the prediction accuracy.

Regarding the choice of quantum model chemistry (basis sets, DFT functionals and wavefunction methods, continuum solvent model and dispersion correction), we would first like to note that in the original manuscript we have described how we systematically tested many methods (geometry optimization and single point energy procedures) and have documented the prediction accuracy obtained for each model chemistry (Supplementary Dataset 5). As mentioned in SI section 2.7 and shown in the Supplementary Dataset 5, we observe that the prediction accuracy is more sensitive to the geometry optimization procedure than to the details of the single point energy (SPE) model chemistry choices: including D3 dispersion correction during the geometry optimization step results in an improvement in accuracy when testing on the category G3 experimental dataset. On the other hand, the predictions are robust to the single point energy model, as can be seen by the fact that both the DLPNO-based potentials and the doubly-hybrid DFT potentials have similar accuracies (Fig S1). Thus, future improvements should focus on optimizing geometry optimization modeling choices to further maximize accuracy.

**3. Construction of dataset of experimental standard redox potentials**

We use the NIST Thermodynamics of Enzyme-Catalyzed Reactions Database [[22]](https://paperpile.com/c/iMTqC0/ZcWKn) and Robert Alberty’s database of standard Gibbs formation energies [[1]](https://paperpile.com/c/iMTqC0/k9fI) to compile a dataset of 105 standard redox potentials. We note that our approach here follows closely that of Bar-Even et al. (2012) [[23]](https://paperpile.com/c/iMTqC0/toY3J). The resulting dataset of standard redox potentials is used to test the accuracy of and calibrate our quantum chemistry predictions.

The NIST database consists of experimental measurements of equilibrium constants for ~450 unique enzymatic reactions, while the Alberty database consists of Gibbs formation energies for ~150 unique metabolic compounds. We use all unique metabolic reactions in TECRDB to build a stoichiometric matrix S_TECRDB/Alberty_, and append additional columns to S_TECRDB/Alberty_ corresponding to the Gibbs formation energies in the Alberty database. We take all possible pairs of compounds appearing in the matrix and compute the difference of their group decomposition vectors, keeping pairs of metabolites (i.e. half-reactions) with group difference vectors that are consistent with the four redox reaction categories (see section “Generation of comprehensive database of natural and non-natural redox reactions”). We then test which of these half-reactions can be obtained from a linear combination of columns in S_TECRDB/Alberty_. In practice this is done by evaluating whether a reaction is orthogonal to the null space of S_TECRDB/Alberty_. We thus keep reactions that are in the span of S_TECRDB/Alberty_. The energetics of the half-reactions that are in the span of S_TECRDB/Alberty_ can then be obtained by simply taking the corresponding linear combination of standard Gibbs reaction/formation energies. We note that in order to span half-reactions with S_TECRDB/Alberty_, we need to add two half-reactions to S_TECRDB/Alberty_, NAD = NADH and NADP = NADPH.

The experimental values contained in TECRDB and the Alberty database come from a variety of sources, with measurements performed in different conditions (pH, ionic strength, temperature, buffer composition). In order to standardize the experimental data for comparison to both GCM and quantum chemistry predictions, we make use of an inverse Alberty-Legendre transform [[24–28]](https://paperpile.com/c/iMTqC0/V7HW7+ktrGL+P0eCn+MV4Xz+0Pdb9) approach to obtain the reaction energies in the pH=7 and I=0.25 standard state. The inverse Legendre transform makes use of known acid dissociation constants to convert the set of available experimentally *apparent* equilibrium constants K’ into a set of standard Gibbs reaction energies ΔG_r_’^o^.

**4. Molecular fingerprint and group contribution method estimates of redox potentials**

**4.1 Group contribution method**

To obtain Group Contribution estimates of redox potentials, we use the group matrix and the group energies of Noor et al. [28[]](https://paperpile.com/c/iMTqC0/uNCQg). The group matrix specifies the group decomposition of each metabolite. Rows of the group matrix correspond to compounds in the KEGG database, while columns correspond to groups. Thus each row corresponds to the group vector $\nu_{g}$ (the decomposition into groups) of a metabolite. To get the GCM estimate for a redox half-reaction, we take the difference between group vectors of products and substrates: $\Delta\nu=\sum_{p_{i}} \nu_{g}(p_{i}) -\sum_{s_{j}} \nu_{g}(s_{j})$. We then take the dot product of this group difference vector and the vector of group energies. Fig S3 shows the predictions obtained with GCM in comparison to calibrated quantum chemistry for each of the four categories. In order to count the number of parameters used by the group contribution method to estimate the redox potentials of each reaction category, we counted the non-zero elements in the group difference vectors $\Delta\nu$of all substrate-redox pairs in a given category.

Similar to the quantum chemical predictions, the GCM estimates were standardized to the E'^m^(pH=7, I=0.25) state. First we obtain the energies from the raw group energy matrix, which correspond to those of the most abundant species at pH=7 (what is sometimes referred to as the pH=7 chemical standard state). We then use the Alberty Legendre transform, which takes into account the pKa’s of substrates and products, and the extended Debye-Huckel equation, and obtain the GCM estimates for the E'^m^(pH=7, I=0.25) values.

**4.2 Molecular Fingerprints**

To compare the prediction errors obtained with quantum chemistry against those of alternative modeling techniques, we implemented a cheminformatics approach to estimating the standard redox potentials of metabolites. We used binary molecular fingerprints to represent metabolites’ structures. Using the RDKit software tool (http://www.rdkit.org), these fingerprints can be obtained directly from the SMILES 40 string representation of each molecule. Because of the relatively small size of our training set (less than one hundred reactant/product pairs and experimental reduction potentials for each reaction category) and in order to avoid overfitting, we decided to use MACCS Key 166 fingerprints instead of other popular binary fingerprints such as Morgan circular fingerprints. We concatenated each redox half-reaction substrate/product fingerprint pair into a single “reaction fingerprint”[[30]](https://paperpile.com/c/iMTqC0/ZyQC9). These reaction fingerprints and their corresponding experimental redox potential values were used as input training data for regularized linear regression.

We performed an independent regression for each of the four different redox reaction categories. We tested both Ridge and Lasso regularization methods as implemented in the scikit-learn machine-learning library for Python[[10]](https://paperpile.com/c/iMTqC0/LNfJe). We used Leave-one-out cross-validation (LOOCV) to evaluate the accuracy of each linear regression model. We optimized the regularization parameter (alpha) required by Ridge and Lasso regularization by performing a grid search procedure. We find that for our experimental data, the optimal values of alpha for Lasso and Ridge regularization are equal to 10-5. In the end, we selected Lasso regression for the downstream analysis based on its higher prediction accuracy as evaluated using LOOCV for categories G2 and G3.

We note however that, given the large number of parameters used by molecular fingerprints despite regularization and the small size of the available experimental data, the molecular fingerprints method is inadequate for estimating redox potentials of categories G1 and G4.

Table S4 shows the accuracies obtained with all three prediction methods: GCM, calibrated quantum chemistry, and molecular fingerprints with regularized regressions. Values in Table S4 correspond to redox potential predictions at the pH=7, I=0.25 state. The number of available experimental values for each reaction category is indicated in parentheses. We consider three different accuracy measures (MAE = Mean Absolute Error, Pearson r, and R2 = coefficient of determination). Note that for the G1 category, quantum chemistry has a lower MAE, but GCM has higher values of Pearson r and R2. Note that for some categories, group contribution method (or molecular fingerprints) result in negative R2 values, indicating that the methods perform worse than a model that always predicts the expectation value of the available experimental data. Categories G1 and G4 have too few experimental values to be adequately modelled using the large number of parameters in the Molecular Fingerprints approach.

**5. Redox potentials of redox cofactors in biochemistry**

Table S1 shows the physiological range of reduction potentials for the major classes of biological electron carriers, as determined by their physicochemical properties and characteristic intracellular concentrations. Sources of data for NAD(P)H: [[31–36]](https://paperpile.com/c/iMTqC0/rYoIW+q5xNq+tEysD+z0hYV+vtiFi+L4oty); for Glutathione and thioredoxin [[37–40]](https://paperpile.com/c/iMTqC0/TlZ5m+LtebK+48FoP+WfWz2); for Quinones and methanophenazines [[41,42]](https://paperpile.com/c/iMTqC0/OjHEA+4eW9K); for Flavoproteins and free flavins; for Ferredoxins: [[43–47]](https://paperpile.com/c/iMTqC0/zm8wM+uhJju+6Up75+clJaE+ERhLn); [[48–51]](https://paperpile.com/c/iMTqC0/9xbHB+ES4TX+q6BBf+0F9ow) and for Cytochromes: [[52–54]](https://paperpile.com/c/iMTqC0/Qks8N+2Ktcx+FPHui)

**6. Generation of comprehensive database of natural and non-natural redox reactions**

To generate our data database of redox reactions, we use a decomposition of all metabolites into functional groups as per the group contribution method of Noor et al. This dataset is available as a matrix of dimension 12439 X 163, with rows corresponding to metabolites and columns corresponding to functional groups. We then take all possible pairs of metabolites from KEGG and compute the difference of their group decomposition vectors, keeping pairs of metabolites with group difference vectors that are consistent with the redox reaction categories. For example, pairs of metabolites in the G1 category will have a group difference vector with a +1/-1 in the element corresponding to an aldehyde/carboxyl functional group respectively. This means that a carboxyl group was removed and an aldehyde added in its place, simulating the conversion of a carbonyl to an aldehyde in G1 reactions. Likewise, G2 reactions will have a group difference vector with +1/-1 corresponding to a hydroxyl/carbonyl functional group. In G4 reactions, the hydroxyl group is simply removed, leaving a carbon, also resulting in a +1/-1 group difference vector. G3 reactions on the other hand, will have a group difference vector with +2/-1 corresponding to the addition of a carbon, a primary amine, and the removal of a carbonyl, respectively. The inclusion of the carbon addition is necessary for the G3 category because the group contribution representation of carbonyls includes a carbon but does not for amines; thus when a carbonyl is removed, a carbon must be added in addition to the amine group to maintain the correct number of atoms. Because of the multiple different representations of functional groups in the group contribution method, each individual reaction from a given category could have different group difference vectors. For this reason, we only looked for vectors that contained +1/-1 for the target functional group change, or in the case of G3, +2/-1.

Using this method we succeeded in generating an initial database of redox reactions. However, additional manual and semi-automated data cleansing was required to get the final version of the database. Compounds with metals were filtered out due to their interference with the group difference vectors, which resulted in the selection of invalid reactions, e.g Nedaplatin → Acetate (G4 reaction)

Additionally, reactions containing generic compounds, as indicated by their designation in KEGG, were removed, given that there were more specific equivalents. An example of this is omega-Hydroxy fatty acid → Glycoaldehyde (KEGG ID: C03547 = C00266)

Use of the group difference vectors also failed to account for the chirality of the metabolites so in some instances stereochemistry was not maintained throughout the reaction. In order to solve this, we applied an additional filter which used the conventions for assigning chirality (R/S, L/D) present in molecule names to match chirality between the substrate and product, for example L-Arabinose → L-Arabitol. In general, reactions in which stereochemistry was changed or gained/lost (determined by the chirality naming conventions) were removed. In certain cases, depending on the redox category, the loss or gain of stereochemistry was valid and thus the reactions were kept; This was commonly found in categories G2 and G4, i.e reduction of a ketone to a hydroxyl and the reduction of a hydroxyl, in which the stereochemistry around the carbon is gained and lost respectively, e.g Diacetyl → (R)-Acetoin (G2).

Sugars proved to be especially problematic as those reactions did not maintain stereochemistry throughout; for these reactions, the above filtering method did not suffice, often keeping incorrect reactions such as L-Xylonate → L-Arabinose. For this, we used molecular naming conventions to eliminate the wrong reactions. For example, in G2 reactions in which sugars are converted to alcohols, we would identify the suffixes -ose → -ol, while also ensuring the names of the substrate and product were similar enough, e.g L-Arabinose → L-Arabitol.

A portion of the database included reactions with structural isomers that passed through the initial pipeline, but would not be legitimate reactions. This is a result of the group difference vector method’s inability to specify the exact connection of atoms. Reactions in which structural isomers were present, such as 3-Hydroxy-L-glutamate → 3-Aminopentanedioate, were removed from the database manually.

Finally, the pipeline could not identify reactions which involved sugar ring opening and closing. The reactions of this kind that we do have in our database were added in from already known reactions. We are currently working on a method involving SMARTS to address this problem.

We emphasize here that these manual and semi-automated cleansing steps do not stem from any non-rigorous definition of our reaction categories. Rather, they generally stem from the manner in which the structures of metabolites are stored in the KEGG database, with the most common issue being that the stereochemistry of many compounds in KEGG is often not specified in full detail.

**Summary: Generation of comprehensive database of natural and non-natural redox reactions**

- Using the group contribution method, we generated pairs of metabolites with group difference vectors consistent with their redox category
  - Categories G1, G2, and G4, had group difference vectors of +1/-1 while the G3 category had a group difference vector of +2/-1
- Additional manual and semi-automated filtering as needed:
  - Remove reactions with generic compounds
  - Remove compounds with metals due to their interference with the group difference vectors
  - Ensure that stereochemistry of metabolites is maintained throughout the reaction using stereochemistry naming conventions; The exception being cases in which stereochemical information is lost or gained during the reaction
  - Eliminated incorrect sugar reactions by using sugar naming conventions
  - Manually removed structural isomers as the group difference vector method did not contain atomic connection information.
  - Ring opening and ring closing reactions needed to be added in from previously known reactions. Group difference vectors could not detect such reactions

**References**

1. [Alberty RA. Thermodynamics of Biochemical Reactions. John Wiley & Sons; 2005.](http://paperpile.com/b/iMTqC0/k9fI)

2. [Jinich A, Rappoport D, Dunn I, Sanchez-Lengeling B, Olivares-Amaya R, Noor E, et al. Quantum chemical approach to estimating the thermodynamics of metabolic reactions. Sci Rep. 2014;4: 7022.](http://paperpile.com/b/iMTqC0/mzspx)

3. [Simons J. Molecular anions. J Phys Chem A. 2008;112: 6401–6511.](http://paperpile.com/b/iMTqC0/kXdZ9)

4. [Neese F. The ORCA program system. WIREs Comput Mol Sci. John Wiley & Sons, Inc.; 2012;2: 73–78.](http://paperpile.com/b/iMTqC0/g7A6N)

5. [Becke AD. Density‐functional thermochemistry. III. The role of exact exchange. J Chem Phys. American Institute of Physics; 1993;98: 5648–5652.](http://paperpile.com/b/iMTqC0/toS0w)

6. [Klamt A, Schüürmann G. COSMO: a new approach to dielectric screening in solvents with explicit expressions for the screening energy and its gradient. J Chem Soc Perkin Trans 2. The Royal Society of Chemistry; 1993;0: 799–805.](http://paperpile.com/b/iMTqC0/zIIPz)

7. [Grimme S, Antony J, Ehrlich S, Krieg H. A consistent and accurate ab initio parametrization of density functional dispersion correction (DFT-D) for the 94 elements H-Pu. J Chem Phys. 2010;132: 154104.](http://paperpile.com/b/iMTqC0/dembK)

8. [Becke AD, Johnson ER. Exchange-hole dipole moment and the dispersion interaction revisited. J Chem Phys. 2007;127: 154108.](http://paperpile.com/b/iMTqC0/r6Clg)

9. [Goldberg RN, Tewari YB. Thermodynamics of the disproportionation of adenosine 5’-diphosphate to adenosine 5'-triphosphate and adenosine 5'-monophosphate. I. Equilibrium model. Biophys Chem. 1991;40: 241–261.](http://paperpile.com/b/iMTqC0/pKAZR)

10. [Pedregosa F, Varoquaux G, Gramfort A, Michel V, Thirion B, Grisel O, et al. Scikit-learn: Machine Learning in Python [Internet]. arXiv [cs.LG]. 2012. Available:](http://paperpile.com/b/iMTqC0/LNfJe) <http://arxiv.org/abs/1201.0490>

11. [Perdew JP, Burke K, Ernzerhof M. Generalized Gradient Approximation Made Simple. Phys Rev Lett. 1996;77: 3865–3868.](http://paperpile.com/b/iMTqC0/MrOtX)

12. [Perdew JP, Ernzerhof M, Burke K. Rationale for mixing exact exchange with density functional approximations. J Chem Phys. American Institute of Physics; 1996;105: 9982–9985.](http://paperpile.com/b/iMTqC0/7SJV3)

13. [Zhao Y, Truhlar DG. The M06 suite of density functionals for main group thermochemistry, thermochemical kinetics, noncovalent interactions, excited states, and transition elements: two new functionals and systematic testing of four M06-class functionals and 12 other functionals. Theoretical Chemistry Accounts: Theory, Computation, and Modeling (Theoretica Chimica Acta). Springer; 2008;120: 215–241.](http://paperpile.com/b/iMTqC0/RuhvD)

14. [Staroverov VN, Scuseria GE, Tao J, Perdew JP. Comparative assessment of a new nonempirical density functional: Molecules and hydrogen-bonded complexes. J Chem Phys. American Institute of Physics; 2003;119: 12129–12137.](http://paperpile.com/b/iMTqC0/xtvv6)

15. [Chai J-D, Head-Gordon M. Systematic optimization of long-range corrected hybrid density functionals. J Chem Phys. 2008;128: 084106.](http://paperpile.com/b/iMTqC0/weYKF)

16. [Yanai T, Tew DP, Handy NC. A new hybrid exchange–correlation functional using the Coulomb-attenuating method (CAM-B3LYP). Chem Phys Lett. 2004;393: 51–57.](http://paperpile.com/b/iMTqC0/EhOl0)

17. [Grimme S. Semiempirical hybrid density functional with perturbative second-order correlation. J Chem Phys. 2006;124: 034108.](http://paperpile.com/b/iMTqC0/AeYza)

18. [Kozuch S, Martin JML. DSD-PBEP86: in search of the best double-hybrid DFT with spin-component scaled MP2 and dispersion corrections. Phys Chem Chem Phys. 2011;13: 20104–20107.](http://paperpile.com/b/iMTqC0/uSEsx)

19. [Riplinger C, Neese F. An efficient and near linear scaling pair natural orbital based local coupled cluster method. J Chem Phys. 2013;138: 034106.](http://paperpile.com/b/iMTqC0/PoSUY)

20. [Grimme S, Goerigk L, Fink RF. Spin-component-scaled electron correlation methods. WIREs Comput Mol Sci. John Wiley & Sons, Inc.; 2012;2: 886–906.](http://paperpile.com/b/iMTqC0/jku9c)

21. [Schwabe T, Grimme S. Towards chemical accuracy for the thermodynamics of large molecules: new hybrid density functionals including non-local correlation effects. Phys Chem Chem Phys. 2006;8: 4398–4401.](http://paperpile.com/b/iMTqC0/Hj2RS)

22. [Goldberg RN, Tewari YB, Bhat TN. Thermodynamics of enzyme-catalyzed reactions--a database for quantitative biochemistry. Bioinformatics. 2004;20: 2874–2877.](http://paperpile.com/b/iMTqC0/ZcWKn)

23. [Bar-Even A, Flamholz A, Noor E, Milo R. Thermodynamic constraints shape the structure of carbon fixation pathways. Biochim Biophys Acta. 2012;1817: 1646–1659.](http://paperpile.com/b/iMTqC0/toY3J)

24. [Alberty RA. Equilibrium compositions of solutions of biochemical species and heats of biochemical reactions. Proc Natl Acad Sci U S A. 1991;88: 3268–3271.](http://paperpile.com/b/iMTqC0/V7HW7)

25. [Alberty RA. Inverse Legendre Transform in Biochemical Thermodynamics: Illustrated with the Last Five Reactions of Glycolysis. J Phys Chem B. American Chemical Society; 2002;106: 6594–6599.](http://paperpile.com/b/iMTqC0/ktrGL)

26. [Alberty RA, Goldberg RN. Standard thermodynamic formation properties for the adenosine 5’-triphosphate series. Biochemistry. 1992;31: 10610–10615.](http://paperpile.com/b/iMTqC0/P0eCn)

27. [Noor E, Bar-Even A, Flamholz A, Lubling Y, Davidi D, Milo R. An integrated open framework for thermodynamics of reactions that combines accuracy and coverage. Bioinformatics. 2012;28: 2037–2044.](http://paperpile.com/b/iMTqC0/MV4Xz)

28. [Noor E, Haraldsdóttir HS, Milo R, Fleming RMT. Consistent estimation of Gibbs energy using component contributions. PLoS Comput Biol. 2013;9: e1003098.](http://paperpile.com/b/iMTqC0/0Pdb9)

29. [Noor E, Haraldsdóttir HS, Milo R, Fleming RMT. Consistent estimation of Gibbs energy using component contributions. PLoS Comput Biol. 2013;9: e1003098.](http://paperpile.com/b/iMTqC0/uNCQg)

30. [Schneider N, Lowe DM, Sayle RA, Landrum GA. Development of a novel fingerprint for chemical reactions and its application to large-scale reaction classification and similarity. J Chem Inf Model. 2015;55: 39–53.](http://paperpile.com/b/iMTqC0/ZyQC9)

31. [Bar-Even A, Noor E, Flamholz A, Buescher JM, Milo R. Hydrophobicity and charge shape cellular metabolite concentrations. PLoS Comput Biol. 2011;7: e1002166.](http://paperpile.com/b/iMTqC0/rYoIW)

32. [Albe KR, Butler MH, Wright BE. Cellular concentrations of enzymes and their substrates. J Theor Biol. 1990;143: 163–195.](http://paperpile.com/b/iMTqC0/q5xNq)

33. [Heineke D, Riens B, Grosse H, Hoferichter P, Peter U, Flügge UI, et al. Redox Transfer across the Inner Chloroplast Envelope Membrane. Plant Physiol. 1991;95: 1131–1137.](http://paperpile.com/b/iMTqC0/tEysD)

34. [Bekers KM, Heijnen JJ, van Gulik WM. Determination of the in vivo NAD:NADH ratio in Saccharomyces cerevisiae under anaerobic conditions, using alcohol dehydrogenase as sensor reaction. Yeast. 2015;32: 541–557.](http://paperpile.com/b/iMTqC0/z0hYV)

35. [Zhao Y, Hu Q, Cheng F, Su N, Wang A, Zou Y, et al. SoNar, a Highly Responsive NAD+/NADH Sensor, Allows High-Throughput Metabolic Screening of Anti-tumor Agents. Cell Metab. 2015;21: 777–789.](http://paperpile.com/b/iMTqC0/vtiFi)

36. [Zhang J, ten Pierick A, van Rossum HM, Seifar RM, Ras C, Daran J-M, et al. Determination of the Cytosolic NADPH/NADP Ratio in Saccharomyces cerevisiae using Shikimate Dehydrogenase as Sensor Reaction. Sci Rep. 2015;5: 12846.](http://paperpile.com/b/iMTqC0/L4oty)

37. [Millis KK, Weaver KH, Rabenstein DL. Oxidation/reduction potential of glutathione. J Org Chem. American Chemical Society; 1993;58: 4144–4146.](http://paperpile.com/b/iMTqC0/TlZ5m)

38. [Schafer FQ, Buettner GR. Redox environment of the cell as viewed through the redox state of the glutathione disulfide/glutathione couple. Free Radic Biol Med. 2001;30: 1191–1212.](http://paperpile.com/b/iMTqC0/LtebK)

39. [Kranner I, Birtić S, Anderson KM, Pritchard HW. Glutathione half-cell reduction potential: A universal stress marker and modulator of programmed cell death? Free Radical Biology and Medicine. 2006;40: 2155–2165.](http://paperpile.com/b/iMTqC0/48FoP)

40. [Holmgren A, Morgan FJ. Enzyme reduction of disulfide bonds by thioredoxin. The reactivity of disulfide bonds in human choriogonadotropin and its subunits. Eur J Biochem. 1976;70: 377–383.](http://paperpile.com/b/iMTqC0/WfWz2)

41. [O’Brien PJ. Molecular mechanisms of quinone cytotoxicity. Chem Biol Interact. 1991;80: 1–41.](http://paperpile.com/b/iMTqC0/OjHEA)

42. [Welte C, Deppenmeier U. Bioenergetics and anaerobic respiratory chains of aceticlastic methanogens. Biochim Biophys Acta. 2014;1837: 1130–1147.](http://paperpile.com/b/iMTqC0/4eW9K)

43. [Stiefel EI, George GN. Ferredoxins, hydrogenases, and nitrogenases: Metal-sulfide proteins. Bioinorg Chem. University Science Books Mill Valey, CA; 1994; 365–453.](http://paperpile.com/b/iMTqC0/zm8wM)

44. [Martín AE, Burgess BK, Stout CD, Cash VL, Dean DR, Jensen GM, et al. Site-directed mutagenesis of Azotobacter vinelandii ferredoxin I: [Fe-S] cluster-driven protein rearrangement. Proc Natl Acad Sci U S A. 1990;87: 598–602.](http://paperpile.com/b/iMTqC0/uhJju)

45. [Pereira MM, Jones KL, Campos MG, Melo AMP, Saraiva LM, Louro RO, et al. A ferredoxin from the thermohalophilic bacterium Rhodothermus marinus. Biochim Biophys Acta. 2002;1601: 1–8.](http://paperpile.com/b/iMTqC0/6Up75)

46. [Przysiecki CT, Meyer TE, Cusanovich MA. Circular dichroism and redox properties of high redox potential ferredoxins. Biochemistry. American Chemical Society; 1985;24: 2542–2549.](http://paperpile.com/b/iMTqC0/clJaE)

47. [Luchinat C, Capozzi F, Borsari M, Battistuzzi G, Sola M. Influence of Surface Charges on Redox Properties in High Potential Iron-Sulfur Proteins. Biochem Biophys Res Commun. 1994;203: 436–442.](http://paperpile.com/b/iMTqC0/ERhLn)

48. [Anderson RF. Energetics of the one-electron reduction steps of riboflavin, FMN and FAD to their fully reduced forms. Biochimica et Biophysica Acta (BBA) - Bioenergetics. 1983;722: 158–162.](http://paperpile.com/b/iMTqC0/9xbHB)

49. [Barman BG, Tollin G. Flavine-protein interactions in flavoenzymes. Thermodynamics and kinetics of reduction of Azotobacter flavodoxin. Biochemistry. 1972;11: 4755–4759.](http://paperpile.com/b/iMTqC0/ES4TX)

50. [Gomez-Moreno C, Choy M, Edmondson DE. Purification and properties of the bacterial flavoprotein: thiamin dehydrogenase. J Biol Chem. 1979;254: 7630–7635.](http://paperpile.com/b/iMTqC0/q6BBf)

51. [Ghisla S, Massey V. Mechanisms of flavoprotein-catalyzed reactions. Eur J Biochem. 1989;181: 1–17.](http://paperpile.com/b/iMTqC0/0F9ow)

52. [Dutton PL, Wilson DF, Lee C-P. Oxidation-reduction ptoentials of cytochromes in mitochondria. Biochemistry. American Chemical Society; 1970;9: 5077–5082.](http://paperpile.com/b/iMTqC0/Qks8N)

53. [Cramer WA, Whitmarsh J. Photosynthetic Cytochromes. Annu Rev Plant Physiol. Annual Reviews; 1977;28: 133–172.](http://paperpile.com/b/iMTqC0/2Ktcx)

54. [Munro AW, Lindsay JG. Bacterial cytochromes P-450. Mol Microbiol. 1996;20: 1115–1125.](http://paperpile.com/b/iMTqC0/FPHui)

**SI Figures**

**
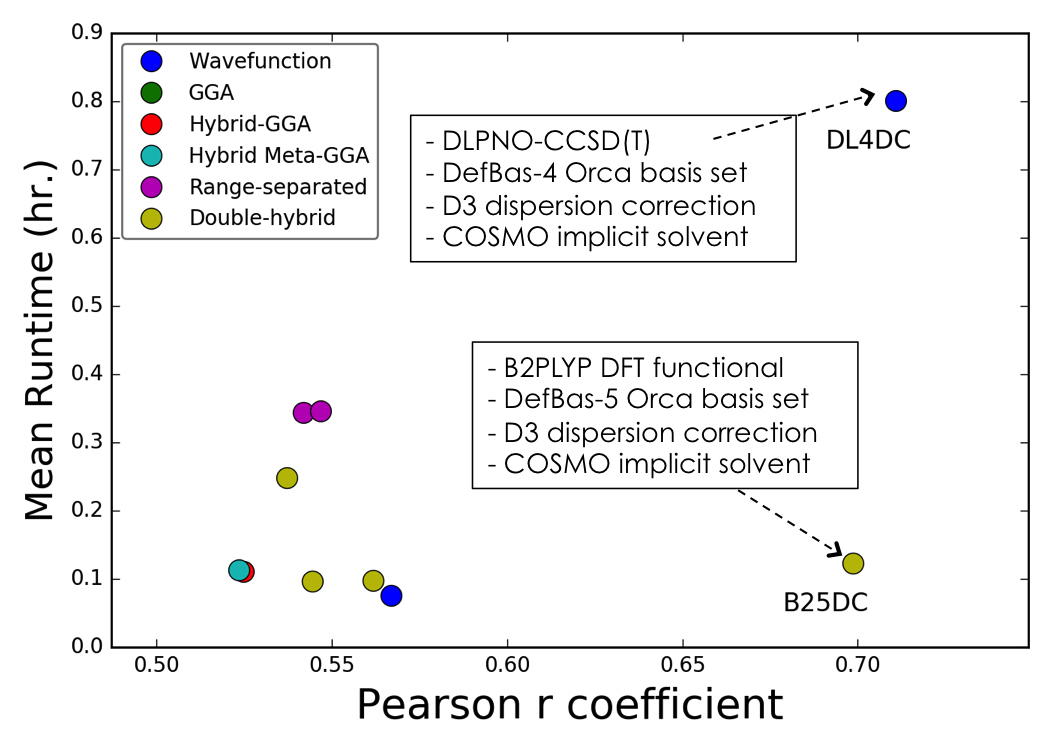
**

**Fig S1** Prediction accuracy, as measured using Pearson r coefficient, and average runtimes per molecular conformer for different quantum single point energy (SPE) model chemistries. The accuracy measures is obtained from comparing the predicted $E'^{m}(pH=7,I=0.25)$ values against available experimental data. Data corresponds to prediction accuracy on the G3 reaction category, which consists of reductions of carbonyls to amines. Mean runtime is calculated over all molecular conformers involved in the simulation of the G3 reaction set with available experimental data. As detailed in section 2.7 “Systematic model chemistry exploration to optimize prediction accuracy”, the SPE model chemistries were obtained from searching over a subspace of possible model chemistries generated from selecting a DFT (or wave function method), a basis set, an implicit solvent model, and a dispersion correction from a total set of: 10 different DFT functionals and 2 wave-function methods, 3 possible basis sets, the option of adding the Conductor-like Screening Model (COSMO) for implicit solvation, as well as the D3 dispersion correction. See Supplementary Dataset 5 for detailed model chemistry descriptions. The option of including or excluding DFT-D3 dispersion correction in the geometry optimization procedure was also considered.


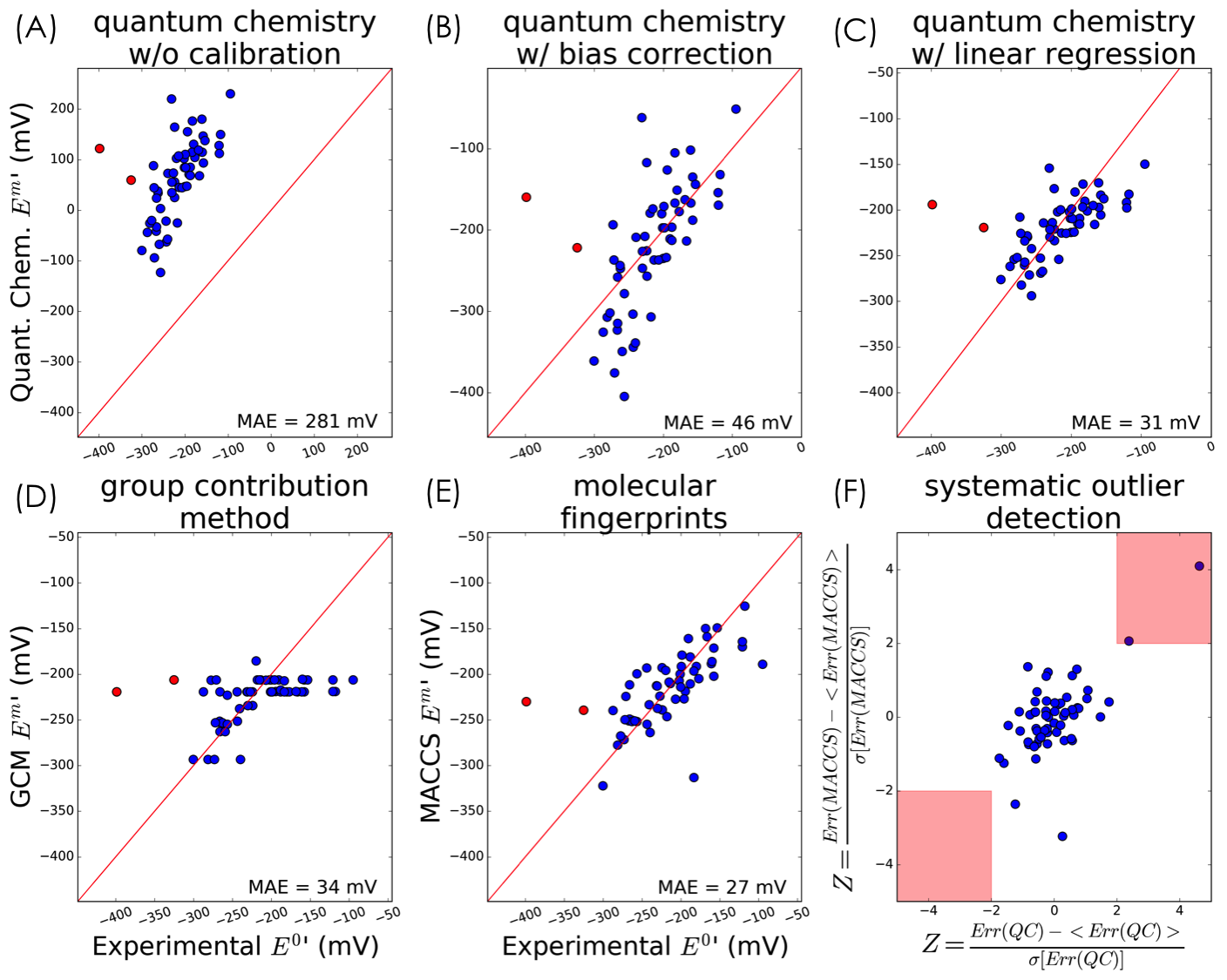


**Fig S2:** Predicting biochemical redox potentials of carbonyl to hydroxycarbon reactions (category G2) with different approaches. (A-C) Calibrating quantum chemical estimates through linear regression (2-parameters per reaction category) significantly improves prediction accuracy. Quantum chemical predictions were performed using the double-hybrid DFT functional B2PLYP, the DefBas-2 Orca basis set, COSMO implicit solvent, and D3 dispersion correction (SI text). Points in red correspond to reactions which consistently appear as outliers across modeling approaches: the indolepyruvate reduction to indolelactate and succinate semialdehyde reduction to 4-hydroxybutanoate (D-E) Prediction accuracy of group contribution method (10 parameters for the G2 category) and molecular fingerprints (166 parameters calibrated with regularized Lasso regression). (F) Scatter plot of normalized prediction errors (z-scores) of G2 reactions for molecular fingerprints and quantum chemistry. The indolelactate dehydrogenase (EC 1.1.1.110) and the succinate semialdehyde reductase (EC 1.1.1.61) reactions have potentially erroneous experimental values.


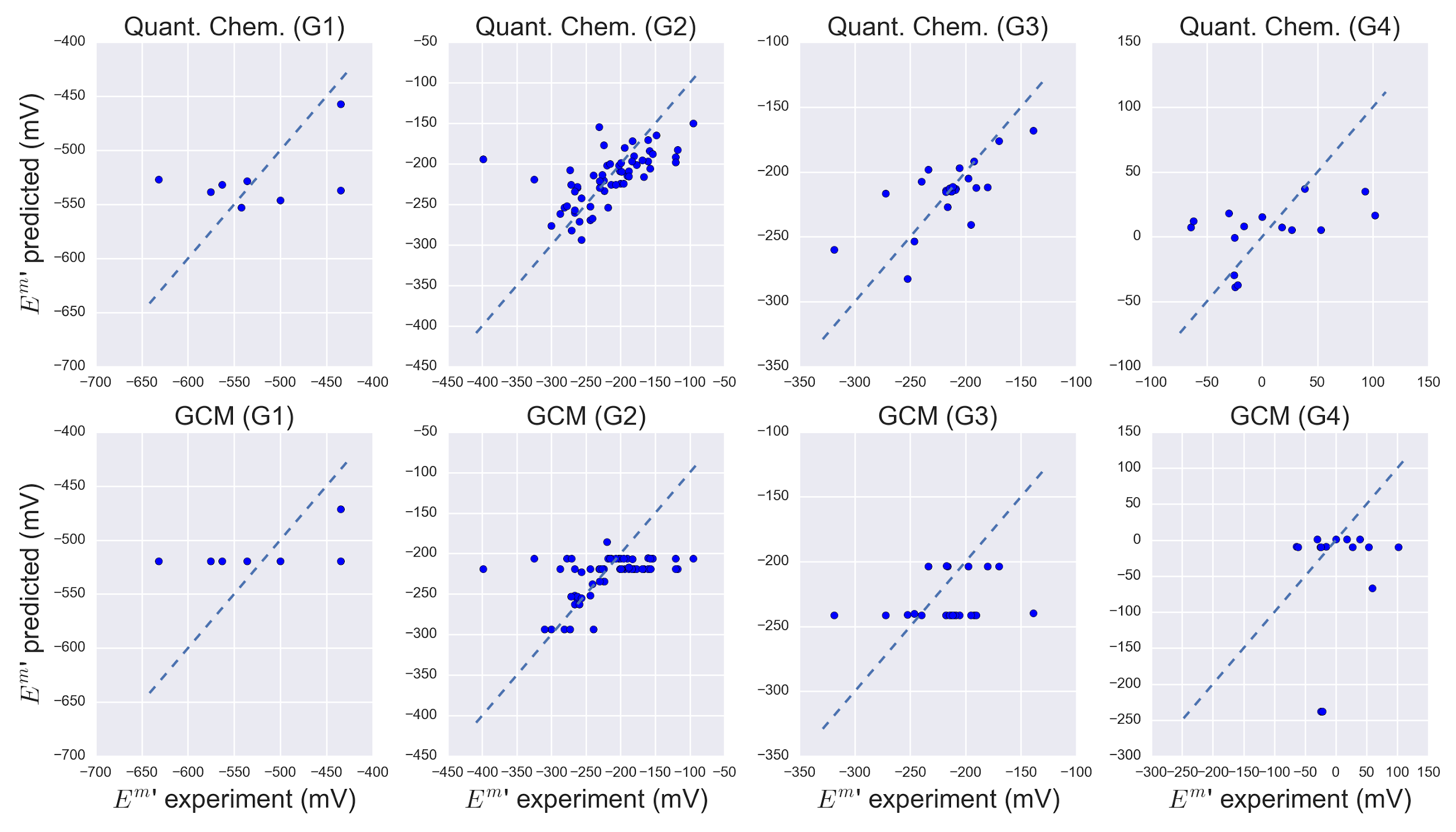


**Fig S3** Scatter plots of experimental redox potentials and predicted potentials with the selected calibrated quantum chemistry approach (upper four panels) and group contribution method (GCM) (lower four panels) for all four redox categories. Quantum chemical predictions were performed using the double-hybrid DFT functional B2PLYP, the DefBas-2 Orca default basis set, the COSMO implicit solvent, and D3 dispersion correction (SI text). Data corresponds to experimental values and predictions at the pH=7 and I=0.25 biochemical state. G1: reduction of an unmodified carboxylic acid (-COO) to a carbonyl (-C=O); G2: reduction of a carbonyl to a hydroxycarbon (-COH, i.e., alcohol); G3: reduction of a carbonyl to an amine (-CNH3); and G4: reduction of a hydroxycarbon to a hydrocarbon (-C-C-).

**
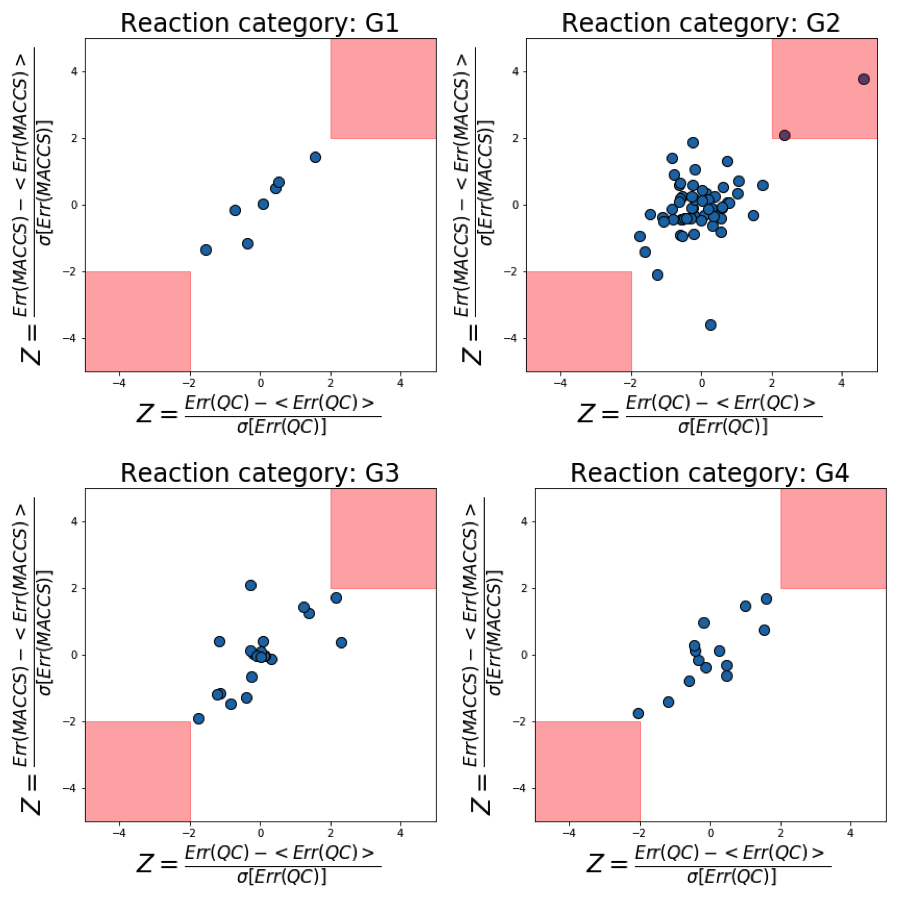
**

**Fig S4:** detection of experimental outliers using a calibrated quantum chemistry approach and MACCS fingerprint predictions for all four reaction categories.

**
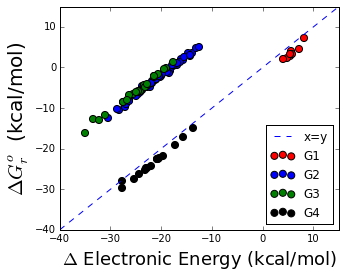
**

**Fig S5:** Correlation between quantum chemical estimates of $\Delta E_{Electronic}$and $\Delta G_{r}'^{o}.$ Each redox reaction category is shown in a different color. G1 - reduction of carboxyl to aldehyde; G2 - reduction of carbonyl (ketone or aldehyde) to hydroxyl; G3 - reduction of carbonyl to amine; G4 - reduction of hydroxyl to hydrocarbon. $\Delta E_{Electronic}$ was obtained from single point energy (SPE) calculations, while $\Delta G_{r}'^{o}$ is obtained by additionally including rovibrational contributions to Gibbs formation energy.


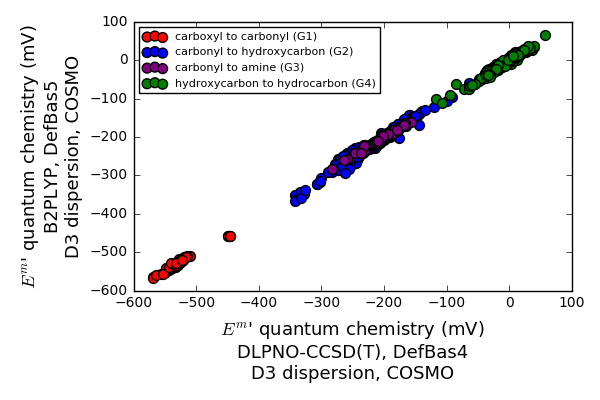


**Fig S6.** Correlation between standard transformed redox potential predictions (pH=7, I=0.25) using calibrated quantum chemistry with our top-two model chemistries. As discussed in the SI text, the prediction accuracy of the calibrated model chemistries was evaluated using the experimental data for the G3 reaction category only (to avoid overfitting). The labels refer to the quantum model chemistry used to perform a single point energy (SPE) calculation on geometry-optimized conformers. For both SPE model chemistries, geometry optimizations were performed using B3LYP functional, Orca’s predefined DefBas-2 basis set (Table S3), COSMO implicit solvent model and DFT-D3 dispersion correction.


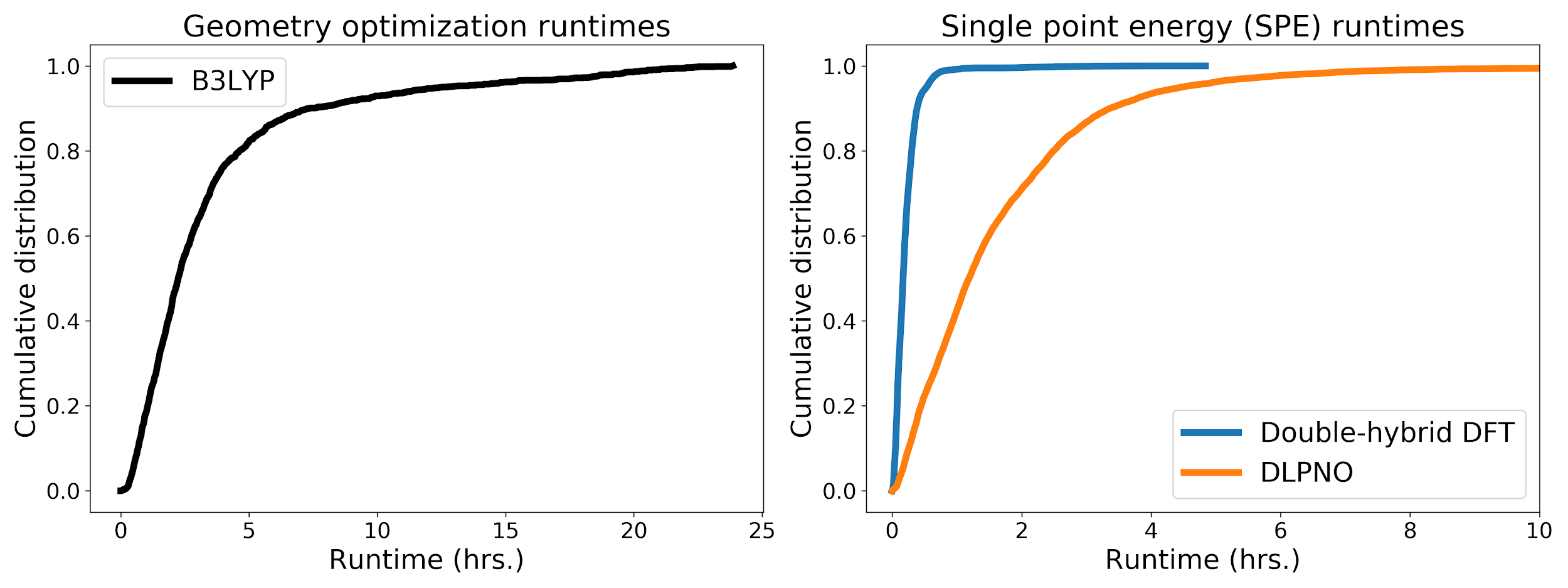


**Fig S7.** Cumulative distribution functions of runtimes for geometry optimization and single point energy (SPE) estimates using our quantum chemistry method. Distributions are over the entire set of molecular conformers used in our study. Geometry optimizations were performed out using DFT, with the B3LYP functional and Orca’s predefined DefBas-2 basis set, as well as the COSMO implicit solvent model (see SI section 2.3). The cumulative distributions of SPE runtimes are shown for the two best-performing model chemistries: the linear-scaling coupled cluster method DLPNO-CCSD(T), with the DefBas-4 Orca basis set (Table S3), COSMO, implicit solvent, and D3 dispersion correction; and the double-hybrid functional B2PLYP, the DefBas-5 Orca basis set (Table S3 for detailed description), COSMO implicit solvent, and D3 dispersion correction (see SI section 2.7 for further details).
